# Inferences from COVID-19 post-exposure risk assessment of health care workers in the pre-vaccination era at a major COVID sentinel center, Sri Lanka

**DOI:** 10.1371/journal.pgph.0001161

**Published:** 2023-02-15

**Authors:** Dammalage L. Bhagya Piyasiri, Wijayawardhene Ganaka G. Senaratne, Pathirawasam Krishantha Jayasekera, Vigeetha H. Withanage, Nayomi S. Danthanarayana, Harshanie A. Ubeysekara, Kankanamge D. Dulnie S. Wijeweera

**Affiliations:** 1 Microbiology, Teaching Hospital Karapitiya, Galle, Sri Lanka; 2 Medicine, Teaching Hospital Karapitiya, Galle, Sri Lanka; 3 Virology, Teaching Hospital Karapitiya, Galle, Sri Lanka; 4 Administration, Teaching Hospital Karapitiya,Galle, Sri Lanka; PLOS: Public Library of Science, UNITED STATES

## Abstract

Despite the hospital triage system trying to prevent COVID-19 patients from getting admitted to wards other than isolation/quarantine units, COVID-19 patients were accidentally being discovered time to time from non-COVID-19 wards due to atypical or asymptomatic presentations. Consequently, post-exposure risk assessment was carried out for the relevant health care workers (HCW) and the other patients to assess their risk level of acquiring COVID-19, and to quarantine them if concluded as high risk. Hence, the objective of the study was to assess the outcome and the adequacy of COVID-19 post-exposure risk assessment of health care workers which would be useful in future outbreaks. We studied all events of accidental detection of COVID-19 patients happened in non-COVID-19 wards which were leading to subsequent risk assessment using the 5-questions based tool adapted from the WHO recommendations. The 5 questions discussed the protective measures during face to face meetings or in physical contacts, protective measures during aerosol generating procedures, splashes onto the face, and hand hygiene measures. A retrospective cross-sectional study carried out in the Teaching Hospital Karapitiya, Galle, Sri Lanka, for 4 months covering the second wave of the pandemic. Hospital data base of risk assessments was accessed anonymously and the “yes” or “no” responses to the 5-questions assessment tool were analysed. There were 62 events involving 891 health care workers who underwent post-exposure risk assessment. From the responses the highest score of “yes” was recorded against question 3 (25.7% of total “yes” answers for all questions and 5.8% of total answers for the question number 3) revealing inadequate precautions taken by HCWs in aerosol generating procedures. Hundred and sixty two (18.2%) HCWs were quarantined as high risk and only one became positive for COVID-19 during the quarantine period. Though the 5-question based risk assessment tool effectively helps to identify breaches in infection control during an exposure to a positive COVID-19 patient, it may not be adequate at times as the only tool in deciding the assessee’s risk level.

## Introduction

With more than 607 million confirmed cases and nearly 6.5 million deaths globally, COVID-19 is called as the worst pandemic people saw in 100 years according to many experts [1, 2]. Before the world started to vaccinate the people against COVID-19, adhering to infection control precautions and quarantining the high risk exposed people, were considered crucial in preventing the spread of COVID-19 especially among health care workers who were constantly being exposed to COVID-19 patients.

Teaching hospital Karapitiya, Galle, is a major tertiary-care hospital and was a COVID-19 sentinel center, which transferred all COVID-19 positive patients to specific treatment centers following diagnosis since the beginning of the pandemic up to April 2021. The hospital operated an on-admission well-updated triage system [3, 4] to prevent suspected COVID-19 patients from getting admitted to wards other than isolation/quarantine units. How much the triage was effective [4], COVID-19 patients were accidentally detected from other wards due to several reasons including, surge in the number of COVID patients in the community [5], onset of new symptoms suggestive of COVID-19 whilst in the health care setting, or the discovery of a contact history/exposure history/geographic location of a high-risk area. An accidental COVID-19 patient was defined as “an earlier non-suspected patient or person tested positive for COVID-19 when screened before a surgery or an invasive procedure, or when tested voluntarily”.

Further, there had been very few cases of accidental exposure to COVID-19 positive health care workers (HCWs) including doctors, nurses, medical undergraduates and to COVID-19 positive bystanders/carers as well. In all these events, other health care workers had been exposed to these positive patients and there was an obvious risk for them to acquire the disease nosocomially due to inadvertent errors in the safety precautions. As a result, post-exposure risk assessment [6–8] was carried out in each incidence for the said health care workers and the other patients in the particular ward/unit to decide the risk level of them to acquire COVID-19 following the particular exposure and to quarantine the contacts who were concluded as high risk. Further, this process helped to address the panic and fear among the health care workers and to assure the safety of other non-COVID-19 patients.

## Justification

Health care workers are strictly advised to adhere to the recommended infection prevention and control protocols against COVID-19 illness, both national and institutional. While any deficit in the adherence rendered the exposed-health care worker concluded as belonging to the high risk category, it is important to highlight those common deficits in the practice which can be used as an eye-opener for the entire health care staff.

The HCWs who were concluded as high risk were quarantined for minimum 14 days which created major setbacks in the health services due to the inadequate number of the staff and caused an economic loss to the country as well as to the individuals. Most of the quarantined HCWs did not develop the disease but had to stay in the isolation for the entire recommended duration. So sometimes there were concerns regarding the adequacy of the risk assessment especially in the health care settings where most HCWs adhered to preventive measures as much as possible.

There had been no such analysis of risk assessments done in this country so far and even the studies published overseas are scarce.

Further this analysis covered the 2^nd^ wave of the pandemic and the pre-vaccination era of the health care workers in the country in which the formal risk assessment was very crucial in prevention of nosocomial outbreaks of the COVID-19 disease.

Finally, this analysis might be very useful to prepare a better risk assessment tool with more practical aspects in a potential future pandemic as well.

## Objectives

Our objectives were to analyse the outcome and the adequacy of the risk assessment procedure performed on health care workers who got accidentally exposed to confirmed or suspected COVID-19 patients within the hospital.

## Methodology

### Study design and the setting

**Fig 1:**
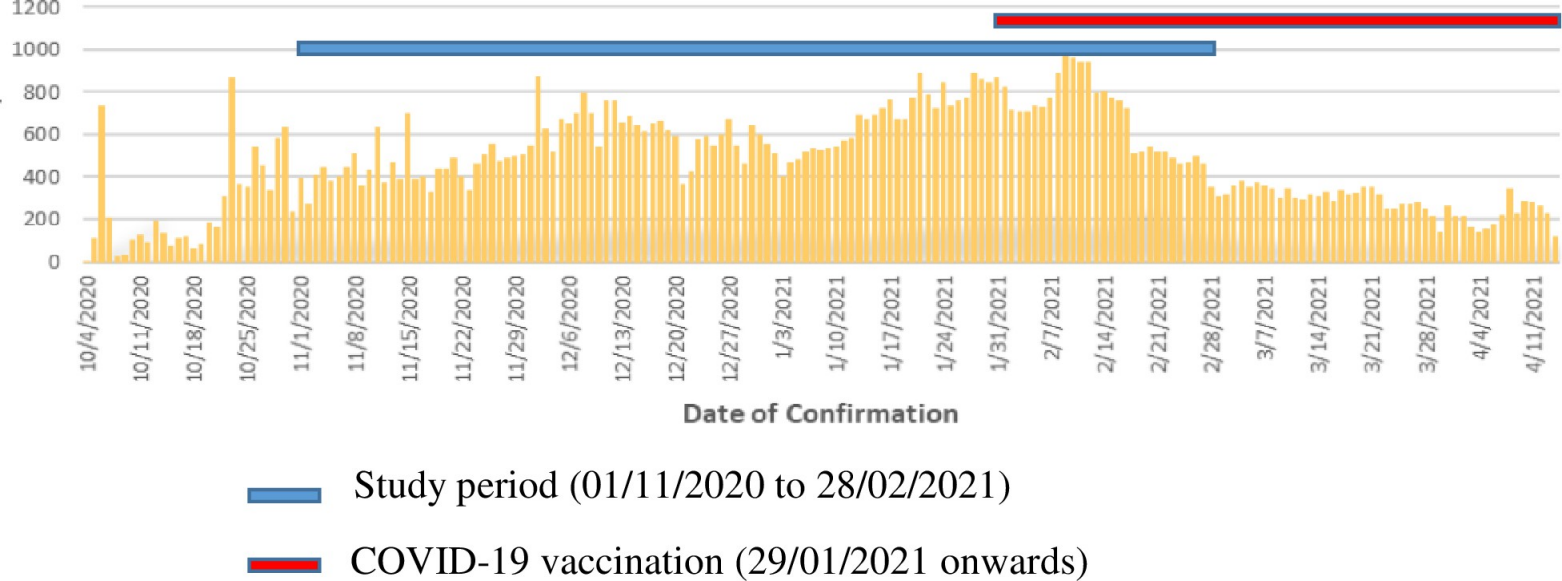
Epidemiological curve: COVID-19 2nd wave, Sri Lanka (04/10/2020 to 4/04/2021) [5].

This retrospective cross-sectional analysis included all the risk assessment events on exposed HCWs which were carried out in Teaching Hospital Karapitiya, Galle, Sri Lanka, from November 2020 to February 2021 for 4 months which we considered as the 2^nd^ wave of the COVID-19 pandemic in Sri Lanka (Fig 1) [5].

The diagnosis of COVID-19 was made by real-time Reverse Transcriptase-polymerase chain reaction (RT-PCR) and rapid antigen test (RAT) in the virology laboratory, Teaching Hospital Karapitiya, Galle.

When a patient was detected accidently from a non-COVID-19 ward (non-isolation/quarantine) of the hospital, the affected team requested the Director of the hospital to conduct the risk assessment and subsequently post-exposure risk assessment was conducted by a team of hospital experts. The team consisted of the consultant microbiologist, consultant virologist and 2 consultant physicians along with the chief nursing officer and a deputy director who coordinated the task.

Risk of each exposed health care worker was assessed according to the 5-questions based-tool issued by the Ministry of Health [6]. It was adapted from the World Health Organization (WHO) [7] and Centers for Disease Control and Prevention (CDC) [8] guidelines (Table 1). The 5 questions consisted of information on transmission based precautions [9] such as appropriate personal protective equipment (PPE), hand hygiene of the HCWs, and the nature of the exposure to the positive patient and patient handled items/environment.

**Table 1:** 5-questions based risk assessment tool.

1. Did you have face-to-face contact (within 1 metre) with a confirmed or probable COVID-19 patient for more than 15 minutes, without you and/or the patient wearing surgical face masks?	
2. Did you have a direct physical contact when providing care to a confirmed or probable COVID-19 patient without wearing appropriate PPE?	
3. Were you present when any aerosol-generating procedures were performed on a confirmed or probable COVID 19 patient, without wearing appropriate PPE?	
4. Was there a splashing of secretions on to the mucus membrane when providing care for a confirmed or probable COVID 19 patient?	
5. Did you have any health care interactions with a confirmed or probable COVID 19 patient without appropriate personal protective equipment (PPE)?	
High Risk	If the answer is **YES** to **ANY** of the above questions for a confirmed COVID 19 patient
Moderate Risk	If the answer is **YES** to **ANY** of the above questions for a probable COVID 19 patient
Low Risk	If the answer is **NO** to **ALL** of the above questions for a probable or confirmed COVID 19 patient And Other situations as indicated by local risk assessments
(Protected exposure)	

The expert panel explained the procedure and the questions in the tool before commencement of the assessment for the HCWs to understand and to be aware of their own risk based on their exposure. End of the assessment the healthcare workers who underwent the assessment, were to fill a form provided by the hospital office, sign, and hand over back to the team if they agree with the decision of the risk assessment team.

According to the COVID-19 post-exposure risk assessment guidelines, a single positive answer for the 5-question tool categorized the assessee as high risk with the recommendation of 14 days quarantine. The panel obtained opinion of the COVID experts team from the Ministry of Health in certain difficult-to assess incidents which will be discussed later.

Analysis of all accidental detection of COVID-19 positive patients was done by utilizing the anonymous data base of laboratory records. The number of HCWs who underwent risk assessment and the subsequent number of HCWs concluded as high risk were also calculated. Also the number of HCWs who became positive for COVID-19 during quarantine was analysed to make a presumptive conclusion regarding the adequacy of the risk assessment procedure.

### Study population

The health care workers who were subjected to the post-COVID-19 exposure risk assessment following accidental exposure to the COVID-19 positive patients in their wards in the teaching hospital Karapitiya, Galle, was the study population.

### Sample size

This was a retrospective analysis of available data and there were no previous studies done on the subject. However, a presumptive sample size was calculated as 384 as follows, assuming that 50% of the exposed health care workers were concluded as high risk following the risk assessment.

n = Z^2^pq/d^2^ (n = = sample size, p = prevalence of high risk HCW taken as 50%, q = (100-p), d = 05 margin of error)

For better results, we analysed all risk assessment events consecutively during the period.

### Inclusion criteria

All responses to the 5-questions based tool during the intended period were included provided the assessment forms submitted by the HCWs had been properly filled and duly signed according to the data base.

### Exclusion criteria

According to the data base, risk assessments outside the study period and data from any incomplete /unsigned forms submitted by HCWs were excluded from the analysis.

### Ethical clearance

HCWs consented by signing the form they filled after the risk assessment that they agreed with the decision of the assessment team and for sharing the details of their responses in the infection control awareness programmes as a part of the service improvement in the hospital. All the forms were duly recorded at the same time as a requirement of the quarantine law of the country and for official use in granting quarantine leave for the staff.

Permission from the hospital administration was taken to access to the said data base which was made totally anonymous prior to access. Only the information of responses to the 5 questions and the category of the health care worker were included in the analysis. Ethical clearance was obtained from the ethical review committee of the faculty of Allied Health Sciences, University of Ruhuna, Sri Lanka, after submitting all relevant documents including the format of the risk assessment form.

### Statistical analysis

The Fisher exact test was used to analyse the association between categorical variables. A *p* value less than 0.05 in a two tailed test was considered as statistically significant.

## Results

We analysed 62 accidental exposure events happened in routine wards involving 891 health care workers who got exposed to 47 patients, 14 health care workers and one bystander who were accidently found positive with COVID-19 infection during the study period.

### Accidently detected patients

There were 47 confirmed COVID-19 patients who were accidentally discovered during this period. Routine pre-procedural testing with RT-PCR or RAT was positive in 23.4% (11/47).

There was a cluster of 17 patients (17/47) in an oncology ward found PCR positive during screening following an accidental identification of a positive patient-carer and was successfully contained, while none of the others caused any hospital clusters.

There were 3 doctors, 4 medical undergraduates, 6 nursing officers, and 1 physiotherapist were found COVID-19 positive with PCR and only 6 HCW infections were nosocomial in origin either from patients (4) or from working colleagues (2).

### Risk assessment

**Fig 2:**
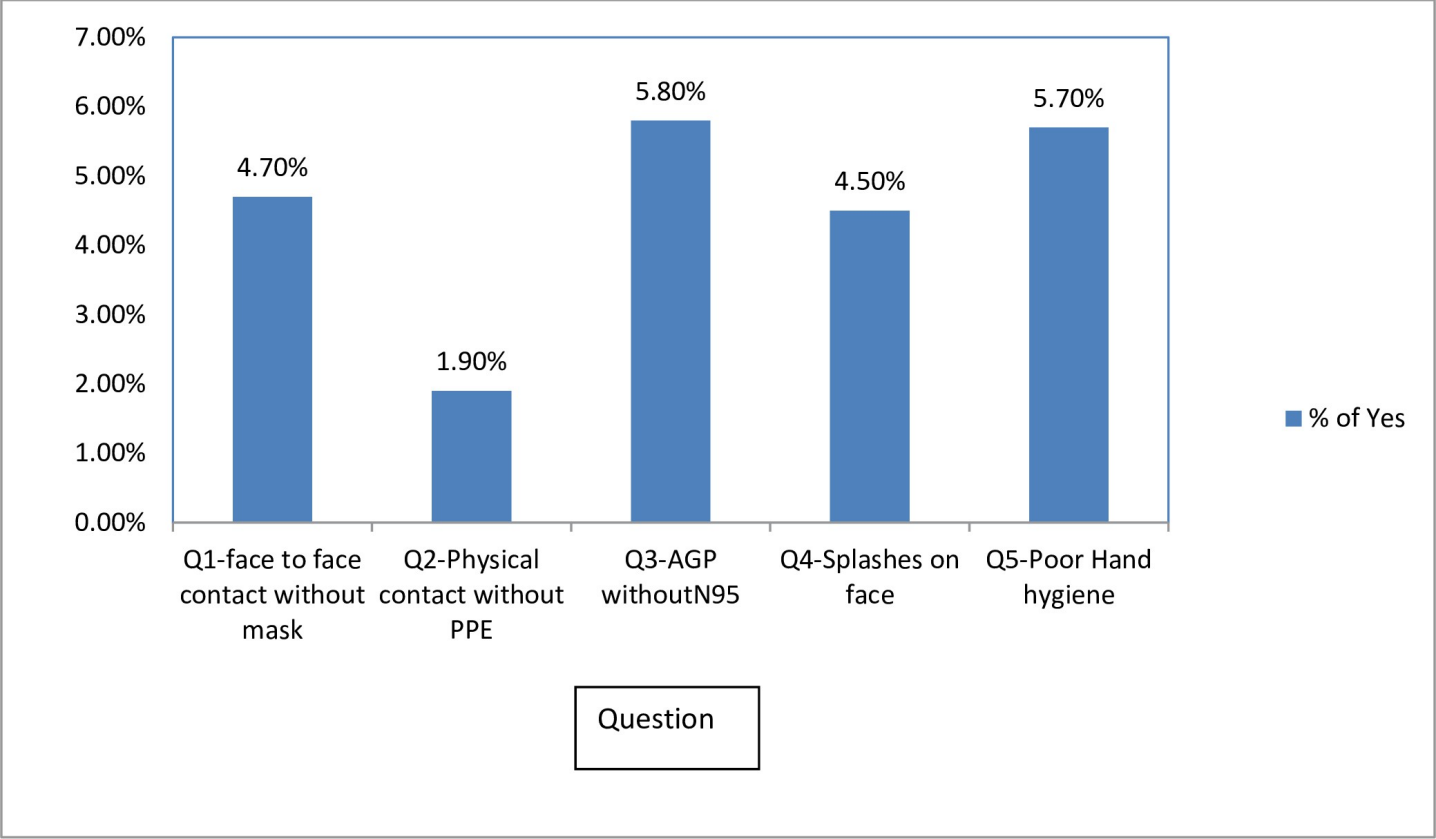
Rate of “yes” responses for each question in the assessment tool.

There were “yes” for each of all 5 questions or for multiple questions with the highest score against question 3 (Fig 2) which resulted in total 202 positive answers. Findings showed that, the most critical mistakes in the clinical setting were observed for questions 3 (25.7% of total “yes” answers) and 5 (51/202, 25.2%) showing major breaches in infection control including violation of 5-moments of hand hygiene rule of WHO [10]. The HCW had performed aerosol generating procedures without N95 masks especially in emergency cardio-pulmonary resuscitation (question 3) and had handled positive patient items without gloves or hand hygiene subsequently (question 5). In case of exposure events to COVID positive co-workers, the HCW had shared rest rooms/tea rooms without wearing masks for more than 15 minutes within 1 meter distance from each other and answered “yes” for question number 1 (42/202, 20.8%). There were 40 instances where the HCW were found without face shield/goggles in which the HCW sustained a splash on eyes from a symptomatic patient especially during emergency cardio-pulmonary resuscitation (question 4, 19.8%). Also, there were few cases where patients were handled without gloves or hand hygiene in-between (question 2, 8.4%).

### Outcome of risk assessment of HCW

**Fig 3:**
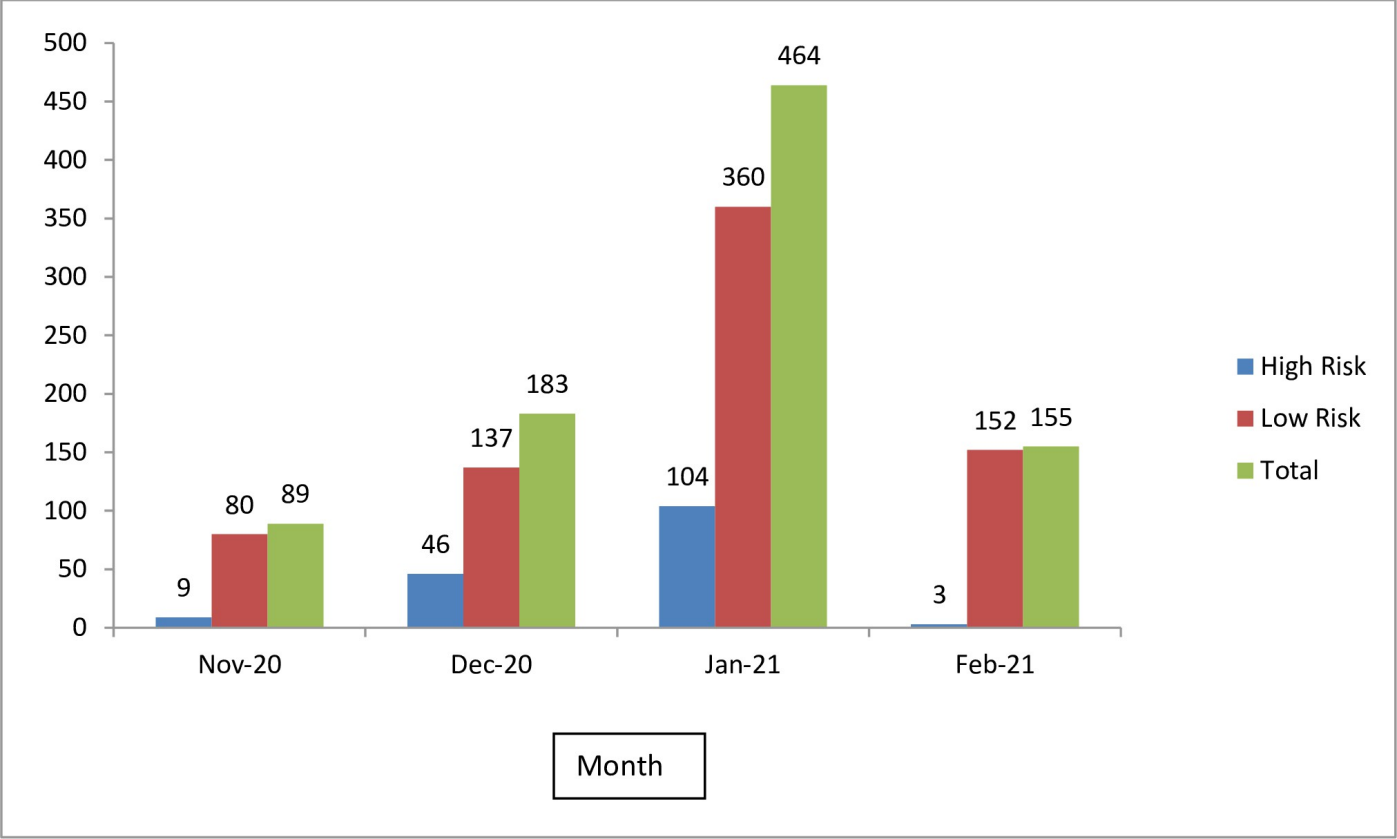
Number of HCWs quarantined as high risk from November 2020 to February 2021.

**Fig 4:**
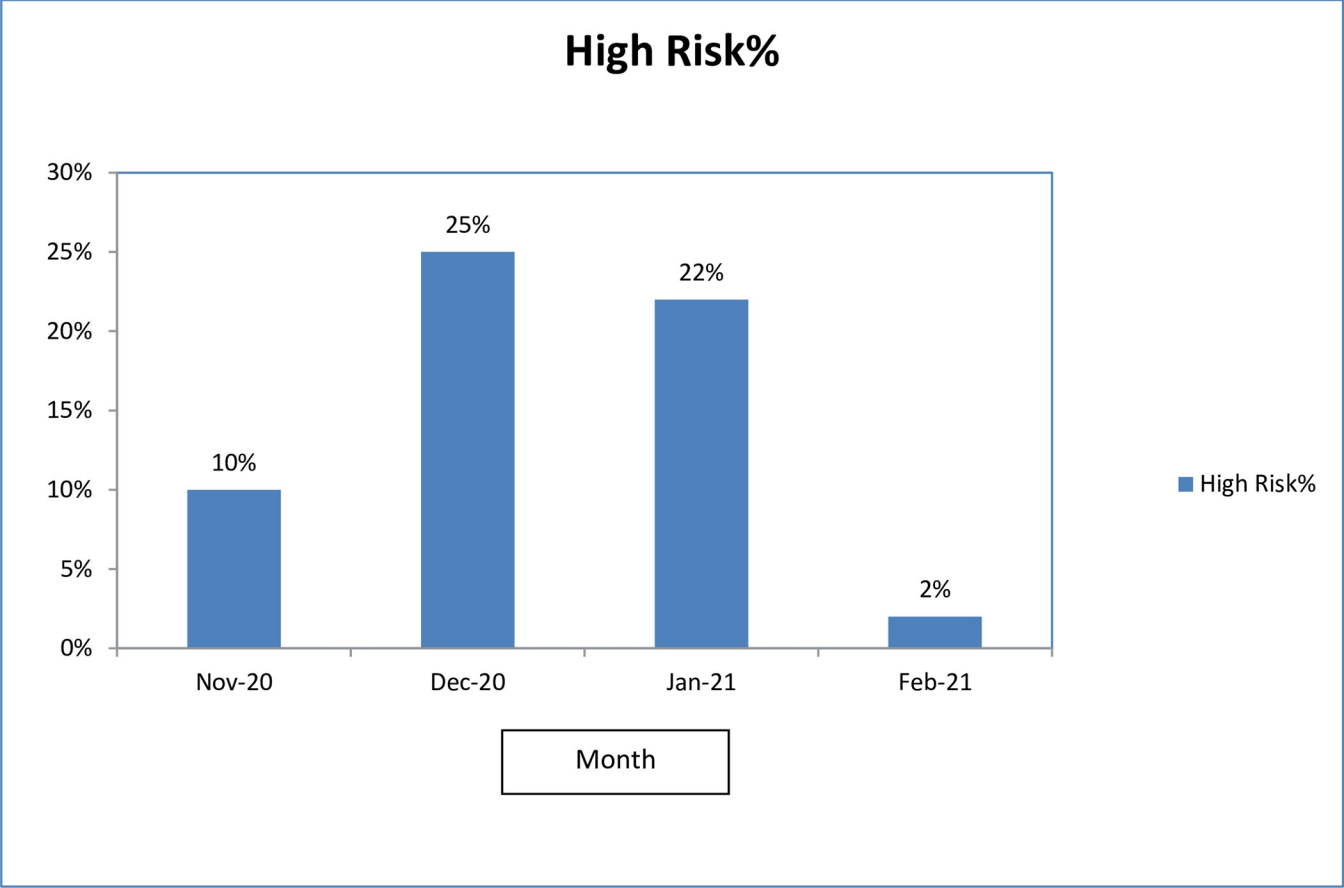
Percentage of HCWs quarantined in each month from November 2020 to February 2021.

Total 891 HCW from the 62 incidents underwent risk assessment during the 4 months. Sixteen incidents resulted in quarantining 162 exposed HCW as having high risk according to the 5-question based assessment by the team of experts (Figs 3 and 4) while 46 incidents involving 729 HCW were concluded as low risk exposures. Those quarantined were 29 doctors including 8 specialists, 104 nursing officers, 4 from paramedical staff, and 25 health assistants during this period. All were quarantined for 14 days and only one became positive with exit PCR who had remained asymptomatic.

### Guidelines on safety measures against COVID-19

Due to non-adherence to and lack of awareness for the previously issued guidelines by January 2021, the team of experts issued 2 concise guidelines on protective measures which included recommendations for AGP, for bystanders/carers, and for safety in rest rooms and tea rooms. The importance of adhering to these guidelines was emphasized in each risk assessment event, in weekly and monthly meetings of the infection control team and in other meetings with different categories of HCW, with the aim of establishing the concept of “protected exposure” among HCW. The idea was not to impair the patient care at any time while being protected with appropriate personal protective equipment and the other infection control measures including hand hygiene.

There were several instances where aerosol generating procedures were done in open wards for accidentally detected patients. Since there was a scarcity of negative pressure chambers, cubicles with full cover were installed in wards where procedures like nebulization or suctioning were performed frequently.

Since 1^st^ February there were only 3 HCW sent on quarantine having assessed as high risk following exposure within the hospital, even we had accidental detection of 12 patients during the month (Figs 3 and 4). The difference of quarantine rates in pre- and post- guideline periods was statistically significant (Fishers exact test-2 tailed, p<0.0001).

## Discussion

### Uncertain facts of risk assessment

It should be accepted that the 5-question risk assessment tool covered almost all the aspects of exposure and helped in the decision of risk level of the exposed HCW independently of the infectivity of the positive index patient. Several studies have shown the value of such risk assessment in preventing the infection being spread among health care workers [11, 12]. However, there were some uncertain areas we experienced with some facts.

When a HCW gives a positive answer to the 5^th^ question on handling patient items and the potential contaminated surfaces without protection, it was difficult for us to assess the significance of that exposure as the question did not define the nature, contact time and frequency of such exposure. Also, it was difficult to assess the significance of using the common toilets shared with a positive patient. This fact was particularly important in assessing the risk level of other patients in the ward. However, we tried to address this problem by allocating one toilet per patient cubicle without causing the whole ward sharing the same toilets. And the staff toilets were decontaminated at least twice per day and particularly after the night shifts.

The risk assessment tool had not considered the infectivity of the patient and we faced uncertainty with PCR positive patients having high Ct values (more than 30). This fact may not be practical in a common guideline as Ct values cannot be directly compared between assays of different types [13]. However, a recent study by Laferl et al from Austria had recommended lifting isolation of the high risk health care workers if the RT-PCR Ct-value of a naso- or oropharyngeal swab sample is over 30, because positive results obtained from genes targeted with Ct-values > 30 correspond to non-viable/noninfectious particles that are still detected by real time RT-PCR [14].

In our assessment, sometimes positive antibody levels in patients with high Ct values were taken into account in deciding the exposure as high or low risk.

### Guidelines and improvement of safety measures

There was a statistically significant reduction of the number of HCWs undergoing quarantine in February 2021. It might have been a result of additional safety measures and the improved awareness addressed by the new institutional guidelines which highlighted the importance of wearing N95 masks in aerosol generating procedures and of hand hygiene according to 5 moments rule of WHO. Reduction of the number of HCW undergoing 14 days compulsory quarantine at that time was immensely helpful to ensure uninterrupted patient-care in the institute at the peak of the second wave of the pandemic (Fig 1).

### Staff quarantining

The most challenging outcome of risk assessment was the interruption of service of a particular unit when its HCWs were sent on quarantine together in large numbers. This issue was addressed by limiting the service only for urgent cases in some special units or by sharing the health care staff from other units in most cases. Except 8 HCWs, all others had chosen to be home isolated rather than opting for institutional quarantine. For all quarantining staff, first PCR or RAT was almost always arranged by the hospital diagnostic service itself and the exit PCR was arranged by the medical officer of health or the public health inspector covering the area of patient location.

### Post-exposure COVID-19 infection during quarantine period

Though 162 exposed HCW were concluded as having high risk only one became positive (0.62%) with exit PCR who had remained asymptomatic. Quarantining them following the post-exposure risk assessment was a precautionary measure to prevent nosocomial outbreaks but the adequacy of it may be seen as an overreaction with a mere 0.6% positivity. However, at the time with less experience of the pandemic and in a pre-vaccination era, it had served its purpose ensuring the physical and mental wellbeing of the staff.

## Conclusions

Since COVID-19 patients can present asymptomatically or with a myriad of symptoms, a high degree of suspicion and protected exposure are the keys to contain the infection, while pre-procedural testing can also contribute to minimize the spread.

Issuing of new guidelines on protective measures and initiation of some other preventive steps during the study period might have caused reduction of high risk exposures even at the height of the 2^nd^ wave.

Though the 5-question based risk assessment tool effectively helped to identify breaches in infection control during an exposure to a positive COVID-19 patient, it may not be adequate at times as the only tool in deciding on high risk and quarantine in cases where the RT PCR Ct values are high.

We would propose incorporation of infectivity of the index case to the risk assessment tool to make it more comprehensive in HCW risk assessment.

## Supporting information

S1 File Ethical approval page 1. (PDF)

S2 File Ethical approval page 2. (PDF)

S3 File Permission from hospital administration. (PDF)

S4 File Risk assessment form-COVID. (PDF)

S1 Data Database on positive answers for high risk. (XLSX)
