# Supplementary material for: Inferences from COVID-19 post-exposure risk assessment of health care workers in the pre-vaccination era at a major COVID sentinel center, Sri Lanka
Source: PLOS Glob Public Health. 2023 Feb 15;3(2):e0001161. doi: 10.1371/journal.pgph.0001161 (PMC10021685; doi:10.1371/journal.pgph.0001161)
Supplement: S2 File — (PDF) [file pgph.0001161.s002.pdf]

Committee requires that you furnish it with progress reports (six monthly) on the study and a final report at the completion of the study, using the appropriate forms at the ERC website, FAHS, UOR. Please report to the ERC any serious adverse events that may occur, in keeping with applicable national regulations and guidelines. If an extension for the period of study is required, it will depend on the progress report submitted and the reason for extension.

Please note that ethical approval will be revoked if any alteration is made to the research protocol without obtaining prior written consent from the ERC.

As the Principal Investigator, you are expected to ensure that procedures performed under the project will be conducted in accordance with all relevant national and international regulations and guidelines that govern research involving human participants.

You are also responsible for negotiating individual arrangements with the heads of service departments in those situations where the use of their resources is involved, or if appropriate, registering the study with a Clinical Trials Registry.

Yours sincerely,

Chairperson
Ethics Review Committee
Faculty of Allied Health Sciences
University of Ruhuna - Galle

....

Date: 12/10/2021

Dr. (Mrs.) HH Peiris
Chairperson, Ethics Review Committee
Faculty of Allied Health Sciences, University of Ruhuna
