# Supplementary material for: Inferences from COVID-19 post-exposure risk assessment of health care workers in the pre-vaccination era at a major COVID sentinel center, Sri Lanka
Source: PLOS Glob Public Health. 2023 Feb 15;3(2):e0001161. doi: 10.1371/journal.pgph.0001161 (PMC10021685; doi:10.1371/journal.pgph.0001161)
Supplement: S3 File — (PDF) [file pgph.0001161.s003.pdf]

Department of Microbiology and Infection Control
Teaching Hospital Karapitiya, Galle
02.07.2021

Director
TH Karapitiya
Galle

Dear Sir,

Permission for data analysis in post-COVID exposure risk assessment

As the expert committee which carries out the post-COVID risk assessment on health care workers, we thought of analyzing the high risk exposures and the deficits in infection control measures which led to the conclusion of high-risk exposure and subsequent quarantine. We believe that the analysis would provide necessary insight on the protective measures against COVID-19 which is of utmost importance in the current pandemic.

Therefore, please be kind enough to give your permission to analyse the data gathered in risk assessment on health care workers for 4 months from November 2020 to February 2021 with a view of subsequent publication as a paper titled **"Experience of the post-exposure risk assessment of COVID-19 exposed health care workers from a major COVID sentinel center, Sri Lanka".**

We ensure the confidentiality of all health care workers involved and analysis would require only the answers provided in the risk assessments.

Study proposal is attached for your perusal.

Thank you
Yours sincerely,

Permitted
07/07/2021

Dr D.L.Bhagya Piyasiri
Consultant Microbiologist (Principal investigator)
Dr W.G.G.Senarathne (Consultant Physician)
Dr P.K.Jayasekara (Consultant Physician)
Dr V.H.Withanage (Act. Consultant Virologist)
Dr N.S.Danthanarayana (Consultant Virologist)
Dr H.A.Ubeysekara (Consultant Community Physician, Deputy Director)
Dr K.D.D.S.Wijeweera (Senior Registrar – Microbiology)

Dr. W.A.M. SHELTON PERERA
Director
Teaching Hospital
Karapitiya
