# Supplementary material for: Inferences from COVID-19 post-exposure risk assessment of health care workers in the pre-vaccination era at a major COVID sentinel center, Sri Lanka
Source: PLOS Glob Public Health. 2023 Feb 15;3(2):e0001161. doi: 10.1371/journal.pgph.0001161 (PMC10021685; doi:10.1371/journal.pgph.0001161)
Supplement: S4 File — (PDF) [file pgph.0001161.s004.pdf]

Risk assessment of COVID-19 post-exposure – TH Karapitiya

Date:-_____/ ____/2020/21 **Unit: - ––––––**

Name: ____

Designation: - ____

QN	Exposure	Yes / No
1	Did you have face to face contact (within I meter) with a confirmed or Probable COVID-19 patient for more than 15 minutes, without you .and /or patient wearing surgical face mask?	
2	Did you have a direct physical contact when providing care to a confirmed or probable COVID-19 patient without wearing appropriate PPE?	
3	Were you present when any aerosol-generating procedures were performed on a confirmed or probable COVID-19 patient, without wearing appropriate PPE?	
4	Was there a splashing of secretions on to the mucus membrane when Providing care for a confirmed or probable COVID-19 patient?	
5	Did you have any health care interactions with a confirmed or probable COVID-19 patient without appropriate personal protective equipment (PPE)?	

I certify that the above information is true

••
Signature of HCW

Level of risk determined according to the ministry circular letter EPID/400/2019 n-cov

Level of risk identified

**1**	**High Risk**	
**2**	**Moderate Risk**	
**3**	**Low Risk**	

Management will be done according to the risk category decided by the committee based on the guidelines given by ministry of health circular letter EPID/400/2019 n-cov

I agree with the above decision

……………………………………..
Signature of HCW

Committee members:-

1. Dr. Harshanie Ubeysekara - _______________________________________
2. Dr. Krishantha Jayasekera - _______________________________________
3. Dr. Ganaka Senaratne - _______________________________________
4. Dr. Bhagya Piyasiri - _______________________________________
5. Dr. Vigeetha Withanage - _______________________________________
6. Ms. M Ranjani - _______________________________________

Incident Form – Following Exposure to a COVID-19 confirmed / suspected patient

A. General Information

Date : ______________________

Name : ____

Date of Birth : ______________________

Sex : ______________________

Permanent Address : ____

Current Address : ____

Telephone No. :______________________

Identity Card No : ______________________

Designation : ______________________

Place of work : ______________________

B. Details of the exposure:

Date: / . /. ..
Time: a.m. /p.m.
Duration: Nature of exposure:
Institution/department/place that the exposure occurred:
Details of the COVID-19 suspected/confirmed patient:

....

C. Type of PPE used – (please tick)

Full PPE (….)
N95 mask (….)
Medical mask (….)

Gloves (….)
Face shield/goggles (….)
No PPE (….)

I confirm that above details are true to my knowledge and agree to share the details of my responses in the infection control awareness programmes.

………………………………………. Date: …………………….
Signature of the HCW
