# Supplementary material for: Inferences from COVID-19 post-exposure risk assessment of health care workers in the pre-vaccination era at a major COVID sentinel center, Sri Lanka
Source: PLOS Glob Public Health. 2023 Feb 15;3(2):e0001161. doi: 10.1371/journal.pgph.0001161 (PMC10021685; doi:10.1371/journal.pgph.0001161)
Supplement: S1 File — (PDF) [file pgph.0001161.s001.pdf]

Ethics Review Committee
Faculty of Allied Health Sciences
University of Ruhuna, Galle, Sri Lanka

E-mail: ethics.fahs@gmail.com

Chairperson
Dr.(Mrs.) Harshini Peiris
BSc, MSc, PhD

Convenor
Dr.(Mrs.) Sewwandi Subasinghe
BSc, MPhil, PhD

Members
Dr.(Mrs.) Thushari Bandara
BSc, MPhil, PhD

Ms. Bimba Wickramarachchi
BSc, MPhil

Ms. Eranthi Weerathunga
BSc, MPhil

Ms. Kumari de Silva
BSc, MPhil

Ms. Vindya Pathiraja
BPharm, MPhil

Dr.(Mrs) Nirmala Rathnayake
BSc, PhD

Dr.(Mrs) Buddhi Fernando
BSc, PhD

External members
Prof. Sampath Gunawardhana
MBBS, PhD

Dr.(Mrs) Gayani Alwis
MBBS, PhD

Indigenous Medicine
Dr. RHSK de Silva
BAMS, BSc, MD(Ayu), Reading PhD

Lawyer
Ms. Ishara Ranapura
LLB

Statistician
Prof. Bilesha Perera
BSc, MSc, PhD

Civil society
Mr. HSM Hisham

11th October 2021

Dr. DLB Piyasiri,
Consultant Microbiologist,
Teaching Hospital,
Karapitiya,
Galle.

Dear Dr Piyasiri,

Re: Reference No: .2021:07.13
Experience of the post-exposure risk assessment of COVID-19 exposed health care workers from a major COVID sentinel center, Sri Lanka by *D L B Piyasiri, WGG Senaratne, P.K. Jayasekera, V.H. Withanage, N.S. Danthanarayana, H A Ubeysekara, K.D.D.S Wijeweera*

Thank you for submitting the above research proposal, which was considered by the Ethics Review Committee, at its meeting of 28th September 2021. We are pleased to inform you that the Ethics Review Committee, Faculty of Allied Health Sciences, University of Ruhuna has granted ethical approval for the Version 3 of the above proposal with the revised title "**Outcome of the post-exposure risk assessment of COVID-19 exposed health care workers at a major COVID sentinel center in Southern province, during the second wave of the pandemic and pre-vaccination era, Sri Lanka**" effective from 11th October 2021 as per details given below.

The following documents were reviewed and approved:

Document	Version No	Date of Submission
Project proposal	3	23/09/2021
Study instrument - Sinhala	3	
Study instrument - English	3	
Study instrument - Tamil	-	
Participant information sheet - Sinhala	3	
Participant information sheet - English	3	
Participant information sheet - Tamil	-	
Participant consent form - Sinhala	3	
Participant consent form - English	3	
Participant consent form - Tamil	-	

We affirm that none of the study team members were present during the decision-making process of the ERC.
